# Controlled Use of Cannabis Among Young Adults in Los Angeles Across Changes in Cannabis Policies

**DOI:** 10.1007/s11469-025-01608-w

**Published:** 2025-12-20

**Authors:** Stephen E. Lankenau, Janna Ataiants, Mark Prince, Ekaterina Fedorova, Bridgid M. Conn, Emily Ansell, Carolyn F. Wong

**Affiliations:** 1 Department of Community Health and Prevention, Dornsife School of Public Health, Drexel University, 3215 Market Street, Philadelphia, PA 19104, USA; 2 Department of Psychiatry and the Behavioral Sciences, Keck School of Medicine, University of Southern California, 2250, Alcazar St # 2200, Los Angeles, CA, USA; 3 Division of Adolescent and Young Adult Medicine, Department of Pediatrics, Children’s Hospital Los Angeles, 4650 Sunset Boulevard, Los Angeles, CA, USA; 4 Keck School of Medicine, University of Southern California, 1975 Zonal Avenue, Los Angeles, CA 90033, USA; 5 Department of Biobehavioral Health, College of Health and Human Development, Penn State University, 214 Biobehavioral Health Building, University Park, PA 16802, USA

**Keywords:** Controlled use, Cannabis, Policy, Young adults, Latent class analysis

## Abstract

The availability of cannabis has increased due to expanding legalization of cannabis across the USA. Controlled use of cannabis — rules by cannabis users that limit use — is a significant but understudied area in the present policy environment, particularly among young adults. A prospective Los Angeles–based cohort aged 18–26 who used cannabis in the past 90 days was assessed during eight survey waves across 9 years. Four discrete waves were analyzed: wave 1 (2014–2015/medical only policy, *n* = 366), wave 4 (2017–2018/transition to adult use policy, *n* = 275), wave 5 (2019–2020/adult use policy, *n* = 241), and wave 8 (2022–2023/adult use policy, *n* = 193). Five rules of controlled cannabis use were used as indicators in a latent class analysis. Two discrete latent classes — Controlled and Uncontrolled — emerged and became more distinct over time. The Uncontrolled class was a majority across all waves. Probabilities for two rules of controlled use — “no school/work” or “no driving” under the influence — increased overtime, and one rule — “stopping cannabis use” — decreased during the transition to legalized adult use. The Controlled class, which consistently practiced more rules, used less cannabis across all waves and had lower problematic cannabis use in waves 1, 4, and 5 compared to the Uncontrolled class.

Access to greater varieties, amounts, and potencies of cannabis has increased dramatically in the USA as more states legalize cannabis for medical and adult use (ElSohly et al., 2016; Nali et al., 2024). In response, researchers have focused on studying and developing interventions to address problematic cannabis use, including dependency and cannabis use disorder (Budney et al., 2019; Sahlem et al., 2018). Research on controlled use of cannabis — rules and practices by cannabis users that limit the use of cannabis — is limited despite the fact that most cannabis users do not become dependent or develop a disorder (Connor et al., 2021; Lapham et al., 2023). Controlled use of cannabis — along with identifying rules that may attenuate cannabis use — is a potentially significant but understudied area in the present policy environment as access to cannabis expands via medical and adult use legalization, particularly among young adults (Fedorova et al., 2025; Lachance et al., 2022; Mennis et al., 2023).

While Becker (1962) formally introduced the concept of controlled use of cannabis in his classic article “Marihuana Use and Social Control,” the concept of controlled substance use is most closely associated with Zinberg (1984), who began his influential research on drug, set, setting, and the controlled use of intoxicants in the 1970s. While best known as a study of opiate users, Zinberg also researched controlled use of “marihuana,” or cannabis, as part of his initial study that began in 1973, during a period of nearly complete cannabis prohibition in the USA. Central to Zinberg’s theory of controlled drug use are features of “setting”: rules or “social sanctions” regarding whether and how a substance should be used (e.g., don’t drive under the influence); and “rituals,” which comprise stylized, prescribed behavior patterns surrounding substance use (e.g., cocktails before dinner). In Zinberg’s (1984) study of controlled use among cannabis users (*n* = 37), qualitative interviews revealed various rituals, such as playing chess or listening to music after consuming cannabis, and rules, such as “use socially” or “have a particular reason to use,” which were often individualized and not always easily articulated. Overall, Zinberg’s study suggests that controlled use behaviors were linked to less frequent substance use and better social functioning. Since then, only a handful of studies have directly reported on rules of controlled use employed by cannabis users.

Roughly 30 years later, Reinarman and Cohen (2007) surveyed rules of cannabis use among adult users in San Francisco (*n* = 266) and Amsterdam (*n* = 216). By this time, cannabis had been legalized in California since 1996 for medical use and decriminalized and available for adult use in the Netherlands since 1976. This study does not identify controlled users in the samples, but rather reports common “rules for regulating cannabis use,” such as not using cannabis during work or study, during the day, or in the morning.

Lau et al. (2015) report on controlled use behaviors (but not rules explicitly) among older adult cannabis users (*n* = 97) in San Francisco, which included medical cannabis patients (*n* = 33). In this qualitative study, examples of controlled use behaviors included: consuming less cannabis due to increased potency; using concentrated cannabis products to reduce consumption of flower; only smoking indica at night and sativa during the day; and taking breaks from consuming cannabis to reduce use and tolerance. Notably, cannabis users did not commonly define benefits of controlled use in terms of frequency or quantity of use, but rather by maintaining normal social functioning and fulfilling roles (e.g., parents, employees, and community members).

Skliamis et al. (2021) reported rules of cannabis use among young adult European cannabis users (*n* = 1225). Common rules included using cannabis “when I’m done with work/study” and never using cannabis “during or before work/study” or “in the presence of children,” which undergird two key constructs pertaining to controlled use: “setting avoidance” and “risk avoidance,” respectively. A main finding is that daily cannabis users were less likely to apply risk avoidance and setting avoidance rules. A key limitation of this study is that factors predicting use of rules (e.g., demographic, health) were not explored or reported.

Though not directly linked to Zinberg’s (1984) conception of controlled use, the Protective Behavioral Strategies for Marijuana Use scale (PBSM) (Pedersen et al., 2016, 2017) captures behaviors potentially associated with the controlled use of cannabis. This scale (either in long 36-item or short 17-item form) assesses strategies generally focused on the harm reduction aspect of cannabis use, including avoiding using cannabis in certain contexts (e.g., before work/school, public places) or limiting the quantity of cannabis use (e.g., buying less cannabis, a predetermined number of hits), so that higher scores represent more frequent use of protective behaviors. Applying the PBSM in different populations of young adults in the USA and Canada, researchers found that context and/or quantity subscales were negatively correlated to cannabis use frequency and/or consequences of use (Jordan et al., 2022; Mian et al., 2021; Florimbio et al., 2024). Limitations of the PBSM include the survey items being tailored to strategies to reduce smoking cannabis flower, and the items do not represent the range of products in the adult-use cannabis market.

Overall, research on rules of controlled cannabis use, while principally initiated over 50 years ago, remains underdeveloped and does not reflect the current high access cannabis environment. This analysis seeks to address past study limitations by assessing controlled use of cannabis in a cohort of young adult cannabis users in Los Angeles followed over 9 years, spanning the shift in policy from medical only and to legalized adult use in California. In this analysis, study outcomes relate to focal points in Zinberg’s study of controlled use of intoxicants, such as frequency of use and problematic cannabis behaviors. Key research objectives of the analysis, which spans four pivotal policy time points (i.e., medical use only environment, transition to recreational, adult-use cannabis sales, and 2–3 years post recreational cannabis legalization), include (1) identifying latent groups or classes of cannabis users based on rules of controlled use behaviors across different policy environments (from medical only to adult use); (2) identifying factors, such as sociodemographic characteristics and baseline health indicators, that predict membership in these classes; and (3) determining the relationship between controlled use and cannabis use outcomes, such as frequency, intensity, quantity, and problematic use, which are identified as top public health concerns in this policy environment at each time point (Baldwin et al., 2024; Hall, 2018).

## Methods

### Participants

Data for this analysis come from the Cannabis Health and Young Adults (CHAYA) longitudinal study, which recruited 366 young adults who used cannabis in Los Angeles, CA, in 2014 (Lankenau et al., 2017a). Enrollment criteria included being aged 18 through 26 years old, either having a current medical cannabis patient recommendation or never being a medical cannabis patient, lived in the metro area of Los Angeles, could read/speak English, and used cannabis in the past 90 days. The past 90-day timeframe was selected since the CHAYA study included an annual survey and follow-up surveys every 3 months or 90 days, which allowed continuous assessment of cannabis use and related behaviors over a 12-month period. Among the 436 people who met eligibility criteria, 366 people (83.9%) agreed to enroll in the study. No statistically significant differences (i.e., age, gender, and race/ethnicity) were found between individuals enrolled and not enrolled in the study (Lankenau et al., 2017b).

The cohort was assessed annually between 2014 and 2023 for a total of eight waves except for a 1-year period between 2018 and 2019 due to a gap in research funding. Participants were surveyed face-to-face between waves 1 and 3, and remotely via a Research Electronic Data Capture survey link for waves 4–8.

The current analysis utilizes four time points or waves from this study, which captures key policy periods: wave 1 (2014–2015/medical use only), wave 4 (2017–2018/transition to adult-use cannabis sales), wave 5 (2019–2020/adult-use cannabis sales), and wave 8 (2022–2023/adult-use cannabis sales). The analytical sample includes only current users of cannabis (i.e., participants who endorsed cannabis use in the past 90 days during those four waves).

The Institutional Review Board (IRB) of Children’s Hospital Los Angeles (CHLA) reviewed and approved the study. All participants were informed of their rights as human subjects verbally and via a consent form. All participants freely consented to join this study.

### Measures

#### Latent Class Indicators

While Zinberg (1984) provided an overall framework for controlled use based upon the constructs of rituals and rules, the theory does not provide specific guidance on operationalizing controlled use. Thus, we selected five items described below from the baseline survey first deployed in 2014 to approximate rules of controlled use based upon salient research literature, i.e., risk avoidance related to family (Pedersen et al., 2016; Skliamis et al., 2021); regulating cannabis use before or during work or study (Reinarman & Cohen, 2007; Skliamis et al., 2021); risk avoidance related to driving a car (Pedersen et al., 2016); stopping cannabis use for a period of time (Lau et al., 2015; Pedersen et al., 2016); and limiting cannabis use to particular times of the day (Lau et al., 2015; Pedersen et al., 2016).

Responses to the following items were coded 1 or 0 (yes/no) to indicate either controlled or uncontrolled use of cannabis, respectively: (1) getting together with members of their family, such as parents, brothers/sisters, or extended family, in the past 90 days, and if yes, whether participants were under the influence of cannabis (yes = 0, no = 1). (2) Attending college, technical school, or professional training in the past 90 days, and if yes, whether participants completed coursework or attended classes under the influence of cannabis. A separate question asked if participants were employed in the past 90 days, and if yes, whether they had gone to work or performed work-related functions while under the influence of cannabis. Valid responses were merged into one item indicating performing work or school responsibilities under the influence of cannabis (yes = 0, no = 1). (3) Driving a car in the past 90 days, and if yes, whether participants drove under the influence of cannabis (yes = 0, no = 1). (4) Stopping cannabis use for a week or more in the past 12 months (yes = 1, no = 0). (5) Time of day participants typically used cannabis in the past 90 days, choosing from the following categories: “morning,” “afternoon,” “evening,” “late night/early morning,” “anytime of day or night.” This item was recoded into “cannabis use at a specific time” and endorsement of the first four categories was coded as “yes,” while endorsement of the “anytime” category was coded as “no” (yes = 1, no = 0).

Subsequently, we refer to these five items approximating rules of controlled use as indicators of controlled use. In the latent class analysis that follows, participants with greater probabilities of these five indicators are defined as “Controlled” whereas participants with lower probabilities are defined as “Uncontrolled.”

#### Covariates

Five sociodemographic and two health-related variables were used as covariates/predictors of latent classes. Sociodemographic covariates included age at baseline, Hispanic ethnicity (yes/no), sex assigned at birth (female/male), age of cannabis use initiation, and annual income, a wave-specific characteristic dichotomized into “$25,000 or less” and “more than $25,000.” Anxiety and chronic pain, which are common health conditions among young adult medical cannabis patients (Ataiants et al., 2021; Lankenau et al., 2017b), were also assessed as wave-specific variables. Anxiety was measured by a six-item anxiety subscale of the Brief Symptom Inventory-18 (BSI-18; Derogatis, 2001), where raw scores are converted into *t*-scores and scores of 63 and above indicate clinical significance. Chronic pain was measured by a positive response to the question asking about experiencing repeated pain in the past 3 months, such as migraine pain or recurrent pain from an old injury.

#### Distal Outcomes

To assess the effects of controlled cannabis use, four cannabis use practices were examined as distal outcomes: (1) quantity of cannabis typically used per week in the past 90 days, dichotomized into “less than 3.5 g” and “3.5 g or more” (equivalent to less and more than 1/8 of an ounce, respectively, which is a common unit for purchasing cannabis) to create two roughly equal groups; (2) number of days of cannabis use in the past 90 days; (3) number of hits (pull off of a pipe, joint, bong, etc.) per day among participants who reported at least one hit in the past 90 days; and (4) problematic cannabis use as measured by the Severity of Dependence Scale (SDS; Gossop et al., 1995; Martin et al., 2006; van der Pol et al., 2013), a 5-item screening tool assessing worries and concerns related to cannabis use, where a score of 4 and above indicates psychological dependence (Martin et al., 2006; van der Pol et al., 2013). The SDS, which we have used in prior analyses (Ataiants et al., 2024; Conn et al., 2024; Fedorova et al., 2022), has been found to be a consistent and reliable indicator of problematic cannabis use (Conn et al., 2024). All outcomes except the quantity of cannabis (categorical) were treated as continuous variables.

### Analysis

The analytical sample for each wave included participants who both remained in the study and used cannabis in the past 90 days. Descriptive analyses in the total sample were conducted using SPSS 29.0. To evaluate changes from wave 1 to wave 8, chi-square tests were applied to categorical variables, and *t*-tests were used for continuous and count variables. Since some participants did not use cannabis or were lost to follow-up after wave 1, we conducted an attrition analysis (see “Results” and Supplemental Table 1) that compared three groups: (1) participants in the analytical sample (who used cannabis in the past 90 days and were retained in wave 4, 5, or 8); (2) those who were retained but did not use cannabis; and (3) those who were lost to follow-up in a specific wave. These three groups were compared across latent class indicators and potential covariates between baseline (wave 1) and the specific wave (4, 5, or 8).

Subsequent analyses were estimated in MPlus, version 8.11 (Muthén & Muthén, 1998–2017). Latent class analysis (LCA), a model-based approach to classification (Nylund et al., 2023a), was used to explore the heterogeneity of indicators of controlled cannabis use. Separate LCAs were conducted for four waves or time points (1, 4, 5, 8) using the five observed indicators of controlled use of cannabis. Class enumeration was performed with-out covariates, starting with a one-class model and increasing the number of classes by one in each consecutive step. To identify the most optimal model, we evaluated the Bayesian Information Criterion (BIC), a reliable fit metric (Chen et al., 2017), where a lower value indicates better model fit. Additionally, the alternative model (with *k* + *1* classes) and the null model (with *k* classes) were compared using the Vuong–Lo–Mendell–Rubin adjusted likelihood ratio test (VLMR-LRT), where a significant *p*-value indicates a better fit of an alternative model compared to the null model. Classification of latent groups was assessed with entropy (overall class separation) and average posterior probabilities (class-specific classification); values closer to 1 indicate better classification.

To examine the longitudinal stability of the latent classes across waves, we conducted a latent transition analysis (LTA) (Nylund-Gibson et al., 2023b). The LTA modeled class membership at each wave and estimated transitional probabilities based on class enumeration obtained in wave-specific LCAs. We assessed one-lag transitions between adjacent waves (wave 1–4, 4–5, 5–8) in the full wave 1 sample (*N* = 366). For participants who did not use cannabis in the past 90 days at a given wave, their indicators were treated as missing. Longitudinal measurement invariance was evaluated by comparing a model in which indicator probabilities were freely estimated across waves with a model in which they were constrained equally, using the log-likelihood difference test with Satorra–Bentler adjustment (Nylund-Gibson et al., 2023b). Since invariance was not supported (see Supplemental Table 2), wave-specific LCAs were retained as the primary analytic approach.

To relate latent classes to covariates, we used the R3Step procedure in MPlus (Muthén & Muthén, 1998–2017), which estimates a logistic regression of class membership on covariates while preserving the latent class measurement model. We then applied the manual three-step BCH procedure (Asparouhov & Muthén, 2014), which allows combining covariates and distal outcomes, to relate latent classes to distal outcomes while accounting for measurement error and preserving the integrity of the latent classes. First, we estimated an LCA model and saved the BCH weights reflecting classification uncertainty. Then, we used those weights to preserve the measurement model and regressed the latent classes and covariates on the distal outcomes. The Wald chi-square test was used to determine the significance of class differences in thresholds for categorical outcomes and intercepts for continuous outcomes.

Missing data was handled using full information maximum likelihood (FIML) estimation in Mplus.

## Results

### Descriptive Characteristics of the Analytical Sample

Participants were 21.23 years old on average (standard deviation [SD] = 2.48), nearly half (45.9%) identified as Hispanic, a third (33.9%) were female, and initiation of cannabis use occurred at 15.24 years old on average (SD = 2.33) at wave 1 (see Table 1). Between waves 1 and 8, the proportion of participants with an annual income over $25,000 increased significantly from 10.1 to 69.5%; moreover, participants with an annual income of over $50,000 increased from 0.6% at wave 1 to 32.8% at wave 8 (data not shown in Table 1). Anxiety and chronic pain levels remained stable between waves 1 and 8. At wave 1, mean days of cannabis use out of 90 equaled 69.05, while average hits per day equaled 23.38 (median = 12, interquartile range = 6–30). A majority of participants (59.6%) consumed 3.5 g or more of cannabis per week and mean SDS score equaled 2.48. By wave 8, mean days of cannabis use decreased significantly, while daily hits, proportion using 3.5 g and more of cannabis per week, and mean SDS scores remained stable.

Regarding controlled use of cannabis indicators, stopping use for 1 week or more in the past 12 months (67.5%) was most common while avoiding work or school under the influence of cannabis (19.1%) was least common at wave 1. By wave 8, proportions avoiding work/school while under the influence and avoiding driving under the influence — two rules indicating greater controlled use — increased significantly. In contrast, proportions stopping use for 1 week or more decreased significantly by wave 8, which is a trend away from controlled use. Two other indicators — using cannabis at a specific time and avoiding family gatherings while under the influence — remained stable.

The sample (current users only within each wave) decreased by 47% from 366 to 194 participants between waves 1 and 8. The final analytical sample at wave 8 is 193 due to one participant having missing data on all controlled use indicators (Table 1). An attrition analysis of participants not retained by wave 8 (i.e., those who remained in the overall sample but did not use cannabis in the past 90 days, and those lost to follow-up) showed a trend towards a higher prevalence of controlled use indicators compared to the analytical sample at wave 1 (Supplemental Table 1). In other words, participants retained in the analytical sample and who reported rules of controlled use at wave 1 declined over time. Regarding potential covariates, participants retained through wave 8 reported significantly higher anxiety levels at wave 1 compared to those who were not retained.

### Class Enumeration

At each wave, models with up to four classes were fit, as no improvement in model fit was observed beyond that point (Table 2). The two-class model showed the lowest BIC and a significant VLMR-LRT for each wave. At wave 5, the VLMR-LRT remained significant for the 3-class model; however, the BIC did not improve relative to the two-class model. Entropy levels, indicating the overall separation of classes, were generally poor but improved from wave 1 to wave 8 and ranged from low (.55) in wave 1 to moderate (.73) in wave 8 for the two-class models.

To assess whether this improvement could be due to attrition, the wave 1 LCA was repeated including only participants retained at later waves. Entropy values were 0.61 for wave 4 retained sample, 0.56 for wave 5, and 0.59 for wave 8, which is comparable to the original wave 1 estimate, suggesting that attrition did not likely influence class separation.

In contrast to entropy, average posterior probabilities in the two-class models were above 0.8 at all time points, indicating adequate classification of individuals into their most likely latent class. Given the exploratory nature of this analysis, a two-class model, with classes labeled as “Controlled” and “Uncontrolled,” respectively, was selected for each wave.

### Characteristics of Latent Classes

**Fig. 1:**
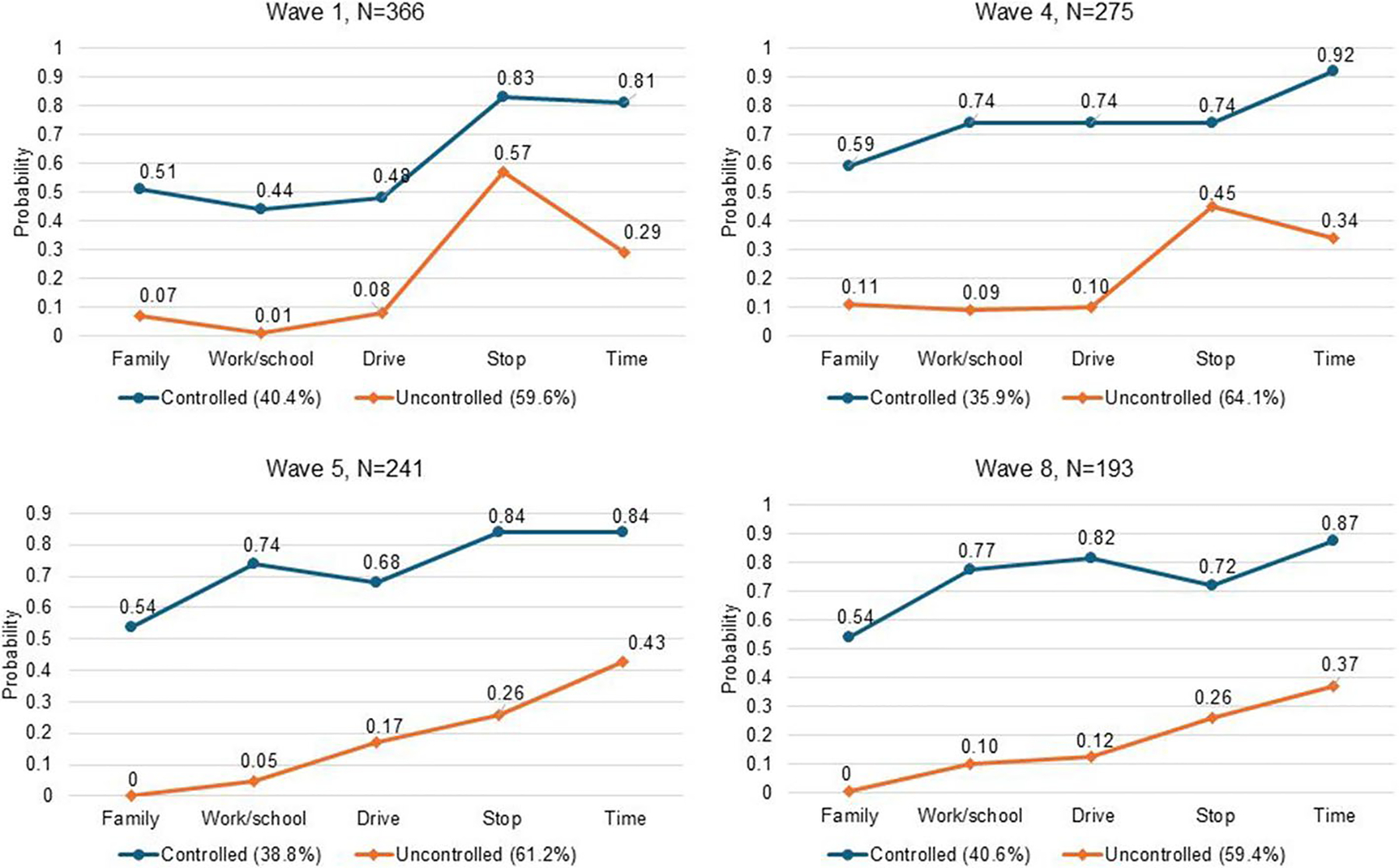
Estimated probabilities of five controlled cannabis use behaviors by latent class (Controlled vs. Uncontrolled) across waves 1, 4, 5, and 8. Latent class indicators include *Family* (avoiding family gatherings while under the influence in the past 90 days), *Work/school* (avoiding work or school under the influence in the past 90 days), *Driving* (avoiding driving under the influence in the past 90 days), *Stop* (stopping cannabis use for a week or more in the past 12 months), and *Time* (using cannabis at a specific time in the past 90 days)

Figure 1, which displays estimated probabilities of indicators of rules of controlled use in each wave, reveals that the Controlled class had higher levels of endorsements of all indicators across all waves compared to the Uncontrolled class. Moreover, the proportion of study participants in the Controlled class declined between waves 1 (40.4%) and 4 (35.9%), which coincided with the start of adult use legalization in 2017, before returning to 40.6% by wave 8. At wave 1, the probability of endorsement of all rules of controlled use was below 0.3 for the Uncontrolled class and above 0.4 for the Controlled class, except for “stopping use at any time,” which had a relatively high probability (above 0.5) among both classes. Among the Controlled class, the probability of all rules increased by wave 8 except for “stopping use at any time.” In particular, probabilities for avoiding cannabis use before work/school and driving increased the greatest in this class between waves 1 and 8. Among the Uncontrolled class, probabilities of rules of controlled use remained consistently low overtime, including “stopping at use at any time,” which declined precipitously between waves 1 and 8.

### Covariates as Predictors of Latent Classes

Table 3 reports the predictors of membership (sociodemographic characteristics and health conditions) in the two latent classes. Later age of cannabis use initiation predicted membership in the Controlled versus Uncontrolled class in both waves 1 and 8 (but not in waves 4 or 5). No other significant differences in sociodemographic or health conditions variables (i.e., ethnicity, sex, age, income, anxiety, or pain) were found between Controlled and Uncontrolled classes at any wave.

### Distal Outcomes of Latent Classes

Table 4 compares Controlled and Uncontrolled classes by assessing key distal outcomes in waves 1, 4, 5, and 8. At wave 1, the Controlled class had significantly lower mean cannabis use days, hits, and a lower probability of consuming 3.5 g or more of cannabis per week as well as a lower mean of problematic cannabis use compared to the Uncontrolled class. This same pattern of significantly lower levels of cannabis use and problematic cannabis use among the classes repeated in waves 4, 5, and 8 — except for problematic use in wave 8.

Earlier, we reported a statistically significant overall decline in mean days of cannabis use (69.1 vs. 61.9) between waves 1 and 8 in the total sample (see Table 1). While statistical comparisons within latent classes across the four time points are not possible due to each participant’s probabilistic membership in all classes, descriptively, estimated mean days of use declined among the Controlled class (49.6 vs. 35.1) but remained stable among the Uncontrolled class (82.4 vs. 81) between waves 1 and 8 (see Table 4).

## Discussion

Zinberg’s (1984) theory of controlled use has renewed relevance in this new era of legalized cannabis, which includes increased access to cannabis and higher potent products. Towards this end, we empirically derived two groups — Controlled and Uncontrolled users of cannabis — based upon five indicators of rules of controlled use of cannabis (Zinberg, 1984; Reinarman & Cohen, 2007; Lau et al., 2015; Pedersen et al., 2016; Skliamis et al., 2021) using data from a longitudinal cohort study of cannabis-using young adults who were recruited in 2014 (Lankenau et al., 2017a) and followed into 2023. Assessing rules of controlled use during the transition from medical only to legalized adult use in California is a particularly novel feature of the study design. At each study time point, the proportion of participants in the Uncontrolled class was greater than the Controlled class. Nonetheless, the Controlled class, over the course of the study, was consistently associated with less cannabis use as well as less problematic use during the first three time points compared to the Uncontrolled class. Based upon these results, a pragmatic public health approach towards reducing cannabis use could include disseminating everyday rules associated with controlled use of cannabis, such as those described in this study.

Importantly, days of cannabis use declined significantly during the transition to legalized adult use in our sample of young adults, which is a trend that we’ve previously reported (Ataiants et al., 2024). Our new results suggest that this overall decline in days of cannabis use could be attributed in some measure to rules of controlled use practiced by participants in the Controlled use class. Such declines in cannabis use post-legalization as reported here mirror a similar trend found in another sample of young adults who frequently or regularly used cannabis prior to legalization (Doggett et al., 2023). However, these findings contrast with general increases in cannabis use among young adults following adult use legalization, but which often focus on prevalence rather than frequency, i.e., days of use, and typically sample from colleges or high school populations (Lachance et al., 2022). Notably, our study design assessed key cannabis constructs, such as days of use and controlled use, 4 years after legalization took effect in California so that our results may reflect the beginnings of longer-term trends rather than immediate after effects of the policy change.

The endorsement of two rules of controlled use — avoiding work/school and driving under the influence of cannabis — significantly increased overtime across the entire sample and corresponded with developmental changes associated with emerging adulthood (e.g., increased responsibilities associated with work and family; Arnett, 2000; Wood et al., 2018). These two indicators also consistently differentiated the two classes (see Fig. 1) and that differentiation is the most pronounced by the final wave. Additionally, the endorsement of another two rules — using at specific times and avoiding family gatherings under the influence — did not significantly change in the overall sample over time but differentiated the two classes consistently across the four time points.

One rule of controlled use — stopping use for 1 week or more — significantly declined overtime across the full sample, which is notable since it suggests that breaks from cannabis use became less common during the policy transition from medical to adult use. While stopping cannabis use for 1 week or more was a consistent feature of the Controlled class, this rule declined precipitously among the Uncontrolled class across the post-legalization period. Declines in stopping cannabis use for 1 week or more among participants could be a function of several factors, such as greater access to cannabis through adult use legalization (Fedorova et al., 2022) and/or rising personal incomes to purchase cannabis, or increased tolerance to cannabis products (Lau et al., 2015).

Apart from the age of cannabis initiation, no other variables were found to significantly predict membership in the Controlled versus Uncontrolled class. In other words, neither sociodemographic factors, such as gender, ethnicity, age, or income, nor health conditions, such as anxiety or pain, were predictive of class membership. The low-to-moderate entropy levels for the latent class models, particularly in wave 1, could be one explanation for this result. Another explanation is that developing and enacting rules of controlled use is a complex and potentially idiosyncratic process that may be driven by variables not accounted for in this analysis, such as other individual, social, or policy factors. Though, later age of cannabis initiation compared to earlier age provides a potential clue since such delayed cannabis initiation is associated with positive outcomes, such as graduating from high school and being employed (Beverly et al., 2019). Thus, later age of initiation could be a marker for other factors predictive of controlled use.

When cannabis use outcomes (i.e., quantity, frequency, intensity of cannabis use, and problematic use) were compared between Controlled and Uncontrolled classes, differences were robust and nearly consistent across all four time points with the exception of problematic use, i.e., SDS, in wave 8. This exception could be a kind of “worried well” phenomenon within the Controlled class as the SDS, which assesses the psychological aspects of cannabis dependence (Gossop et al., 1995; Martin et al., 2006), and may be capturing elevated anxiety about using cannabis itself, despite other behaviors suggesting declining or controlled use. Overall, persons in the Controlled class (i.e., those with higher probabilities of embracing rules of controlled cannabis use) generally used less cannabis and had lower problematic cannabis use scores at each wave compared to the Uncontrolled class. Moreover, as probabilities of enacting rules of controlled use increased at each time point, days of cannabis use decreased. On one hand, this result makes intuitive sense since practicing rules indicative of controlling cannabis use, such as not using at specific times or under specific circumstances, could translate into fewer opportunities to use cannabis (Pedersen et al., 2016). Furthermore, more constraints around cannabis use and fewer opportunities to use cannabis could result in less problems with cannabis (Pedersen et al., 2016). Though, these findings suggest that persons in the Controlled class may take a broader approach to cannabis use than what is represented by the five indicators of rules of controlled use presented in this analysis, which could further explain lower rates of cannabis use and problematic use. For instance, other rules of controlled use not captured in this analysis, such as not using when children are around, using only alone, using only on weekends (Pedersen et al., 2016; Skliamis et al., 2021) may represent other ways that Controlled users modulate their use.

While the current results on controlled use are promising, the fact that roughly two-thirds of study participants fell into the Uncontrolled class is a notable finding. Moreover, participants in the Uncontrolled class consistently reported relatively low probabilities of engaging in rules of controlled use across the four time points of the study, with the exception of declines in stopping cannabis use (indicative of less controlled use), even as these participants aged and potentially achieved developmental milestones (e.g., new jobs, family, role transitions). Overtime, this stability in engaging in few rules of controlled use was linked to a stable, high rate of cannabis use — approximately 80 out of 90 days — across the study period. The lack of findings predicting class membership is important since it leaves few clues as to the characteristics of a person who consistently refrains from employing rules of controlled use. Notably, a participant’s personal income, which is often a potential proxy for career advancement, was not predictive of class membership. At a minimum, this finding suggests that career advancement was not hindered by not embracing certain rules conventionally associated with positive employee behaviors, such as not using cannabis before work (Reinarman & Cohen, 2007; Skliamis et al., 2021), or that some participants may have worked in industries where cannabis use was normative (Reed et al., 2020). Also, non-significant indicators of current health status, such as anxiety and pain, suggest that potentially debilitating health conditions were not driving factors behind not employing rules of controlled use.

### Implications

Zinberg’s (1984) study of controlled use was conducted over 50 years ago during a period of cannabis prohibition in the USA. Throughout earlier periods of cannabis prohibition, declining rates of cannabis use among young adults were often attributed to a maturation process (Chen & Kendal, 1995), where greater responsibilities associated with adulthood, e.g., jobs, family responsibilities, were thought to curb or eliminate substance use (Winick, 1962). A key difference between that period and now is the legality of cannabis, which largely protects adult users from arrest and associated penalties in states where cannabis is legal, and may reduce the need to stop cannabis use altogether for persons concerned about legal consequences. While gradually adopting some rules of controlled use as a young person ages could be viewed as a kind of “maturing out” (Winick, 1962), a key difference is that the Controlled class in this sample still used cannabis at relatively high rates by the end of our study, which coincided with adult use legalization and expanding access to cannabis. Moreover, whereas maturing out is associated with cessation from or reduction in substance use, rules of controlled use limit use in certain circumstances, which in turn are associated with lower frequency and intensity of use, but not stopping use altogether. However, since it is unknown whether young adults who dropped out of this study continued cannabis use or not — and an attrition analysis revealed that a higher proportion who dropped out showed a trend towards controlled use — examining rules of controlled use as a preliminary stage towards cannabis cessation is a potential focal point of future studies. Notably, the proportion of study participants in the Controlled class declined in the years following study enrollment, which overlapped with the policy change of legalization for adult use, but returned to the approximate baseline proportion by the end of the study. While attrition may be a factor in this trend, it is possible that shifting norms related to legalization prompted some participants to loosen their rules for a period of time before reverting back to earlier patterns on controlled use as they continued to assume greater work and social responsibilities by the end of the study.

To reiterate, results demonstrate that some young adults practiced rules of controlled use of cannabis and that the propensity towards certain rules increased overtime as a person aged and matured. This is notable since access to cannabis increased via legalization during this period. Since little is known about how persons learn rules of controlled use of cannabis, there is potential to disseminate and encourage rules of controlled use via public health messaging since rules were associated with lower cannabis use and problematic cannabis use. As a next step, future research should more fully investigate rules of controlled cannabis use beyond the five indicators or rules identified in this analysis. Studying factors that inhibit taking breaks from cannabis use for periods of time, including tolerance breaks (Ansell et al., 2023), may be a particularly important direction.

Despite the potentially productive relationship between rules of controlled use and lower cannabis use and problematic use, it is unclear whether being a member of the Controlled class confers better outcomes than the Uncontrolled class in other key dimensions of functionality, such as happiness or adjustment. In other words, it is possible that persons who rarely employed rules of controlled use and used more cannabis often are as well-adjusted adults as controlled users who consume less cannabis — particularly if they live or work in settings where rules of controlled use are not normalized. As a next step, future studies of controlled use should include broader indicators of overall psychosocial functioning as well as economic well-being to determine whether controlled use of cannabis is linked to other positive health or developmental outcomes. Furthermore, a broader range of predictors of controlled use, including motivations for controlled use, should be identified to gain a better understanding of pathways or determinants of controlled use. Lastly, future studies need to determine how frequently and consistently rules of controlled use are applied in daily life and determine their short-term and longer-term impact more broadly on substance use and health outcomes.

Overall, more public health research is needed that focuses on rules of controlled use of cannabis or other mechanisms, such as cannabis protective behavioral strategies (Pedersen et al., 2016, 2017), associated with reduced cannabis use. Moreover, more research is needed to understand how rules associated with controlled use as described here, e.g., not using cannabis before getting together with family, may differ from protective behavior strategies, e.g., avoiding getting too high, both in terms of mechanisms and outcomes. As legalized cannabis production, sales, and marketing increasingly focus on heightening cannabis consumption (Borodovsky et al., 2021), science-based public health approaches with countervailing messages on how to moderate cannabis consumption are needed.

### Limitations

This study has several limitations. First, between waves 1 and 8 — a 9-year period — the study lost 47% of its wave 1 participants through attrition or stopping cannabis use, which could impact key variables of interest, including variables related to controlled use. However, an attrition analysis revealed that a higher proportion of study participants who dropped out by wave 8 tended to follow the rules of controlled use in wave 1. Hence, study results may be biased in the direction of losing controlled users overtime, which favors a more conservative interpretation of study findings. Second, due to the exploratory nature of this analysis, cannabis forms and modes of use were not examined as outcomes. This is a limitation since potent forms and related modes of administration (e.g., dabbing) — both associated with risky cannabis use — could potentially distinguish controlled users from uncontrolled ones. Third, the “stopping use for 1 week or more” indicator of controlled use could include reasons unrelated to controlled use, such as illness and traveling. However, persons in the controlled group had consistently higher probabilities of “stopping use for 1 week or more” compared to persons in the uncontrolled group, suggesting an overall ability to refrain from using cannabis when needed. Finally, as described above, entropy levels for the latent class models were low-to-moderate, particularly in wave 1, which introduces some uncertainty in class membership classification and underscores the complexity of the controlled use phenomenon. Improving classification precision may require a broader and more diverse set of indicators to capture the multidimensional nature of controlled use. Importantly, entropy improved with each subsequent time point, suggesting a better level of separation among participants as they aged. The ability to better discern latent classes at later waves, when participants were older, may relate to a developmental process where some participants learn to control their use and others do not, and thus, differences between patterns of controlled use become more distinct with age. A sensitivity analysis further indicated that this improvement was unlikely to result from attrition, suggesting that the observed increase in class separation reflects genuine differentiation in controlled use patterns rather than effects of sample retention.

## Conclusion

Rules of controlled cannabis can be identified, quantified, and used to categorize young adults into discrete classes of controlled and uncontrolled cannabis users. In this study, transitions from the medical only to legalized adult use in California coincided with a decline in the proportion of controlled users overall and in one particular rule (i.e., stopping cannabis use in the past 12 months), but increases in other rules (e.g., avoiding work/school or driving under the influence of cannabis). While a later age of cannabis initiation predicted membership in the Controlled versus Uncontrolled class, the study did not reveal any demographic or health-related factors determining class membership. The Controlled class, which is defined by practicing more rules, was associated with lower levels of cannabis use and problematic use across most of the time points of the study compared to the Uncontrolled class. Overall, our study results provide the groundwork for future research into rules of controlled use of cannabis, including understanding different aspects of controlled use informed by setting, and investigating whether disseminating clearly defined rules of controlled use to young adults could lead to public health benefits, such as reduced cannabis use and problematic cannabis use.

## Supplementary Material

Supplemental_Lankenau

**Supplementary Information** The online version contains supplementary material available at https://doi.org/10.1007/s11469-025-01608-w.

## Figures and Tables

**Table 1:** Descriptive characteristics of the sample

Characteristic	Wave 1 (2014–2015)	Wave 4 (2017–2018)	Wave 5 (2019–2020)	Wave 8 (2022–2023)	*p*-value (W1 vs. W8)
	*(N* = 366)	(*N* = 277)	(*N* = 241)	(*N* = 193)	
**Demographic and health covariates**					
*Baseline*					
Age at baseline, mean (SD)	21.23 (2.48)	21.20 (2.46)	21.29 (2.45)	21.42 (2.49)	.38
Hispanic ethnicity, *n*% (*n*)	45.9 (168)	44.0 (122)	45.6 (110)	47.2 (91)	.78
Female sex assigned at birth, % (*n*)	33.9 (124)	35.7 (99)	38.6 (93)	37.8 (73)	.35
Age of cannabis initiation, mean (SD)	15.24 (2.33)	15.30 (2.30)	15.30 (2.26)	15.38 (2.40)	.50
*Wave-specific*					
Annual income (> $25,000), % (*n*)	10.1 (36)	33.2 (82)	53.2 (118)	69.5 (123)	<.001
BSI-Anxiety, mean (SD)	50.32 (9.85)	49.95 (10.88)	52.57 (11.30)	50.96 (11.15)	.50
Recurrent pain 90 d, % (*n*)	32.6 (118)	31.7 (86)	36.1 (84)	35.8 (68)	.45
**Cannabis use outcomes, past 90 days**					
Days of cannabis use, mean (SD)	69.05 (26.64)	59.03 (34.3)	61.68 (32.99)	61.91 (34.08)	.01
Hits per day, mean (SD)	23.38 (26.68)	22.41 (29.47)	20.01 (26.92)	18.48 (27.35)	.05
Median (IQR)	12 (6–30)	9 (4–27)	10 (4–20)	8 (4–15)	
Quantity (≥ 3.5 g per week), % (*n*)	59.8 (217)	60.1 (158)	62.1 (136)	57.5 (100)	.61
SDS, mean (SD)	2.48 (2.71)	2.92 (2.97)	3.13 (3.35)	2.91 (3.34)	.66
**Controlled use behaviors**					
Stopping use for 1 week or more past 12 m, % (*n*)	67.5 (247)	55.4 (148)	47.6 (109)	45.2 (85)	<.001
Using at a specific time past 90 d, % (*n*)	50.0 (183)	54.9 (151)	59.1 (140)	57.5 (111)	.09
Avoiding family gatherings under the influence past 90 d, % (*n*)	24.8 (78)	27.7 (62)	20.8 (40)	21.6 (33)	.45
Avoiding driving under the influence past 90 d, % (*n*)	23.9 (68)	33.6 (73)	37.7 (72)	40.9 (65)	<.001
Avoiding work or school under the influence past 90 d, % (*n*)	19.1 (63)	31.3 (75)	32.7 (67)	38.7 (65)	<.001

*SD*, standard deviation

1) The number of observations for individual variables may differ from the total sample size in each wave due to missing data. 2) p-values in the last column reflect chi-square test results for categorical variables and t-test results for continuous and count variables

**Table 2:** Fit and classification statistics for the latent class models tested

Wave	Number	Classes	BIC	VLMR-LRT	Entropy	Average class posterior probabilities	Class proportion, %
1	366	1	1986				
		2	**1917**	**<.001**	0.55	.85,.91	40.4, 59.6
		3	1941	0.09	0.64	.67–.85	7.4–57.5
		4	1973	0.36	**0.72**	.63–.86	1.4–58.0
4	275	1	1613				
		2	**1504**	**<.001**	0.68	.87,.94	35.9, 64.1
		3	1531	0.21	0.66	.56–.88	13.5–49.4
		4	1556	0.13	**0.84**	.77–.93	4.9–33.2
5	241	1	1374				
		2	**1243**	**<.001**	0.70	.89,.92	38.8, 61.2
		3	1260	**0.04**	0.76	.82–.93	19.2–53.4
		4	1285	0.50	**0.79**	.72–.95	16.1–33.8
8	193	1	1147				
		2	**1035**	**<.001**	0.73	.92,.93	40.6, 59.4
		3	1055	0.12	**0.87**	.90–.95	11.5–58.9
		4	1077	0.06	0.80	.82–.94	15.9–34.6

*BIC*, Bayesian Information Criterion; *VLMR-LRT*, Voung–Lo–Mendell–Rubin adjusted likelihood ratio test Bold font indicates the best fit or classification criteria

**Table 3:** Predictors of membership in Controlled class vs. Uncontrolled class

Wave	Covariate	UOR	95% CI		AOR	95% CI	
1							
	Baseline age	1.02	0.91	1.14	1.04	0.90	1.19
	Hispanic	1.39	0.78	2.48	1.54	0.80	2.95
	Female	0.73	0.39	1.35	0.71	0.36	1.41
	Age of initiation	1.21**	1.06	1.38	1.20**	1.05	1.37
	Income W1	0.45	0.15	1.34	0.48	0.15	1.61
	BSI-Anxiety W1	1.00	0.97	1.03	1.00	0.97	1.04
	Recurrent pain W1	0.73	0.39	1.37	0.75	0.37	1.50
4							
	Baseline age	1.00	0.89	1.14	0.99	0.85	1.15
	Hispanic	0.94	0.51	1.74	0.84	0.43	1.66
	Female	1.15	0.61	2.16	1.17	0.59	2.31
	Age of initiation	1.03	0.89	1.18	1.05	0.89	1.23
	Income W4	0.86	0.43	1.71	0.88	0.42	1.84
	BSI-Anxiety W4	0.99	0.96	1.02	0.99	0.96	1.03
	Recurrent pain W4	0.85	0.44	1.16	0.86	0.41	1.81
5							
	Baseline age	0.95	0.83	1.09	0.91	0.78	1.07
	Hispanic	1.30	0.68	2.48	1.21	0.58	2.54
	Female	1.05	0.54	2.03	1.19	0.59	2.41
	Age of initiation	1.12	0.98	1.29	1.14	0.97	1.33
	Income W5	1.37	0.70	2.68	1.28	0.62	2.66
	BSI-Anxiety W5	0.99	0.96	1.02	0.99	0.96	1.03
	Recurrent pain W5	0.64	0.32	1.29	0.68	0.31	1.49
8							
	Baseline age	1.10	0.96	1.27	1.07	0.88	1.30
	Hispanic	1.24	0.63	2.46	1.97	0.79	4.93
	Female	1.55	0.77	3.12	1.66	0.75	3.70
	Age of initiation	1.29**	1.11	1.51	1.30**	1.09	1.56
	Income W8	0.88	0.41	1.90	0.90	0.38	2.12
	BSI-Anxiety W8	1.01	0.98	1.04	1.01	0.97	1.04
	Recurrent pain W8	1.15	0.56	2.35	0.82	0.33	2.04

*UOR*, unadjusted odds ratio; *AOR*, adjusted odds ratio; *CI*, confidence interval; *BSI-Anxiety*, Brief Symptom Inventory-Anxiety. Sample sizes for the adjusted models are smaller than those reported in Table 1 due to missing data on covariates

** *p* <.01

**Table 4:** Distal outcomes of Controlled and Uncontrolled latent classes

Wave	Distal outcomes	Estimated intercepts/thresholds (SE)		Estimated means/probabilities		Wald *χ*^2^
		Controlled	Uncontrolled	Controlled	Uncontrolled	
1	Quantity (≥ 3.5 g per week)	0.73 (0.24)	− 1.38 (0.24)	0.32	0.80	28.09***
	Days	50.22 (3.25)	82.01 (1.83)	49.57	82.44	54.74***
	Hits per day	12.70 (1.74)	30.70 (2.30)	11.87	31.25	31.61***
	SDS	1.40 (0.21)	3.19 (0.23)	1.44	3.17	26.06***
4	Quantity (≥ 3.5 g per week)	1.18 (0.33)	− 1.40 (0.26)	0.23	0.80	29.78***
	Days	30.39 (4.24)	77.36 (2.30)	30.63	77.21	83.32***
	Hits per day	6.57 (2.11)	30.17 (2.82)	6.29	30.33	37.36***
	SDS	2.34 (0.35)	3.30 (0.24)	2.24	3.36	4.30*
5	Quantity (≥ 3.5 g per week)	0.66 (0.31)	− 1.38 (0.28)	0.34	0.80	18.89***
	Days	32.28 (4.10)	82.20 (2.32)	32.20	82.26	94.75***
	Hits per day	8.81 (2.39)	28.55 (3.10)	8.55	28.72	20.70***
	SDS	2.16 (0.39)	3.66 (0.33)	2.16	3.66	6.80**
8	Quantity (≥ 3.5 g per week)	1.16 (0.40)	− 1.23 (0.28)	0.26	0.77	19.25***
	Days	34.91 (5.06)	81.20 (2.66)	35.14	81.04	52.96***
	Hits per day	10.93 (2.98)	23.07 (3.35)	9.96	23.75	6.12*
	SDS	2.60 (0.52)	3.22 (0.32)	2.40	3.37	0.85

*SE*, standard error; *SDS*, Severity of Dependence Scale (a problematic cannabis use measure). 1) All estimates are adjusted for four time-invariant covariates (baseline age, sex, Hispanic ethnicity, age of cannabis use onset) and three wave-specific covariates (annual income, BSI-Anxiety score, and recurrent pain); all covariates are centered to align model estimates with sample means and proportions. 2) Sample sizes vary due to missing data on covariates. 3) Intercepts and means are estimated for days, hits, and SDS; thresholds and probabilities are estimated for the quantity of use (≥ 3.5 g per week)

* *p* <.05;

** *p* <.01;

*** *p* <.001

## Data Availability

The data that support the findings of this study are available from the corresponding author, [SL], upon reasonable request.
