# Supplementary material for: Controlled Use of Cannabis Among Young Adults in Los Angeles Across Changes in Cannabis Policies
Source: Int J Ment Health Addict. Author manuscript; Available in PMC 2026 Jan 14. (PMC12799194; doi:10.1007/s11469-025-01608-w)
Supplement: Supplemental_Lankenau [file NIHMS2135853-supplement-Supplemental_Lankenau.docx]

**Supplemental Table 1.** The baseline (Wave 1) sample by attrition status at Waves 4, 5, and 8 (N=366)

|  | Wave 1 by attrition at Wave 4 | | | | Wave 1 by attrition at Wave 5 | | | | Wave 1 by attrition at Wave 8 | | | |
| --- | --- | --- | --- | --- | --- | --- | --- | --- | --- | --- | --- | --- |
|  | Retained/90d use (n=277) | Retained/ no 90d use (n=25) | Not retained (n=64) | *p-value* | Retained/90d use (n=241) | Retained/  no 90d use (n=19) | Not retained (n=106) | *p-value* | Retained/90d use (n=193) | Retained/  no 90d use (n=40) | Not retained (n=132) | *p-value* |
| **Controlled use behaviors** |  |  |  |  |  |  |  |  |  |  |  |  |
| Avoided family gatherings under the influence 90d, % | 21.7^a^ | 38.1^a^ | 33.3^a^ | .068 | 20.8^a^ | 52.9^b^ | 29.1^a,b^ | **.007** | 19.8^a^ | 38.2^a^ | 28.3^a^ | **.043** |
| Avoided work or school under the influence 90d, % | 16.3^a^ | 36.4^a^ | 25.0^a^ | **.033** | 15.8^a^ | 33.3^a^ | 23.7^a^ | .074 | 12.9^a^ | 30.6^b^ | 24.6^b^ | **.008** |
| Avoided driving under the influence 90d, % | 20.2^a^ | 40.9^a^ | 32.7^a^ | **.028** | 22.0^a^ | 40.0^a^ | 25.3^a^ | .276 | 17.3^a^ | 40.6^b^ | 28.7^a,b^ | **.008** |
| Stopped use one week or more 12m, % | 66.4^a^ | 80.0^a^ | 67.2^a^ | .381 | 65.1^a^ | 78.9^a^ | 70.8^a^ | .324 | 62.7^a^ | 70.0^a^ | 73.5^a^ | .117 |
| Used at a specific time 90d, % | 48.7^a^ | 80.0^b^ | 43.8^a^ | **.006** | 48.1^a^ | 73.7^a^ | 50.0^a^ | .100 | 47.2^a^ | 62.5^a^ | 50.8^a^ | .207 |
| **Demographic and health covariates** |  |  |  |  |  |  |  |  |  |  |  |  |
| Baseline age, mean | 21.2^a^ | 21.32^a^ | 21.34^a^ | .900 | 21.3^a^ | 19.6^b^ | 21.4^a^ | **.010** | 21.4^a^ | 20.4^a^ | 21.2^a^ | .066 |
| Hispanic ethnicity, % | 44.0^a^ | 48.0^a^ | 53.1^a^ | .412 | 45.6^a^ | 36.8^a^ | 48.1^a^ | .656 | 47.2^a^ | 37.5^a^ | 47.0^a^ | .518 |
| Female sex, % | 35.7^a^ | 28.0^a^ | 28.1^a^ | .415 | 38.6^a^ | 42.1^a,b^ | 21.7^b^ | **.007** | 37.8^a^ | 40.0^a^ | 26.5^a^ | .074 |
| Age of onset, mean | 15.3^a^ | 15.04^a^ | 15.06^a^ | .700 | 15.3^a^ | 14.9^a^ | 15.2^a^ | .689 | 15.4^a^ | 15.1^a^ | 15.1^a^ | .460 |
| Annual income ≥$25,000, % | 9.6^a^ | 4.0^a^ | 14.8^a^ | .276 | 10.0^a^ | 0^a^ | 11.9^a^ | .304 | 11.6^a^ | 2.5^a^ | 10.2^a^ | .222 |
| BSI-Anxiety, mean | 50.13^a^ | 53.09^a^ | 50.14^a^ | .396 | 50.9^a^ | 51.9^a^ | 48.7^a^ | .136 | 51.2^a^ | 51.8^a,b^ | 48.5^b^ | **.033** |
| Recurrent pain 90d, % | 33.8^a^ | 17.4^a^ | 32.8^a^ | .271 | 35.6^a^ | 10.5^a^ | 29.8^a^ | .063 | 35.9^a^ | 30.8^a^ | 28.5^a^ | .360 |

Notes:

1) BSI-Anxiety=Brief Symptom Inventory-Anxiety.

2) The sample size for the “Wave 1 attrition at Wave 8” section is n=365 due to the exclusion of one individual with missing data on all controlled use behaviors at Wave 8.

2) Sample sizes for three controlled use behaviors are smaller due to relevant exposure, including “avoided family gathering under the influence in the past 90 days” (n=315); “avoided work or school under the influence in the past 90 days” (n=330), and “avoided driving under the influence in the past 90 days” (n=284).

3) One-way ANOVA was used to compare means, and the chi-square test of independence was used to compare proportions across retention status. The p-value columns indicate F-test p-values for ANOVA or chi-square p-values; significant p-values (<.05) are bolded.

4) Different superscripts (“a” or “b”) denote proportions that differ significantly from each other at α=0.05.

**Supplemental Table 2.** Model fit, classification quality, and difference test results for noninvariant and invariant LTA models

| **Model** | **LL** | **# Free Parameters** | **Scaling** | **BIC** | **Entropy** | **LRT difference test** | | |
| --- | --- | --- | --- | --- | --- | --- | --- | --- |
|  |  |  |  |  |  | **χ^2^** | **df** | **p-value** |
| Noninvariant LTA | -2764.514 | 46 | 1.0830 | 5801 | 0.71 | 123.6 | 26 | <.001 |
| Invariant LTA | -2826.307 | 20 | 1.2257 | 5771 | 0.72 |  |  |  |

LTA = latent transition analysis; LL = log-likelihood; MLR = maximum likelihood estimator with robust standard errors; Scaling = scaling correction factor for MLR;

BIC = Bayesian Information Criterion; df = degrees of freedom; LRT = log-likelihood ratio test.

Note: The log-likelihood difference test was calculated following the procedure described by Nylund-Gibson et al. (2023b). The chi-square statistic incorporates the Satorra–Bentler scaling adjustment for nested models estimated with MLR. A significant test result (p < .001) indicates that the constrained model fits significantly worse than the freely estimated model, meaning that longitudinal measurement invariance was not supported.
